# Zoom-Delivered Empowered Relief for Chronic Pain: Observational Longitudinal Pilot Study Exploring Feasibility and Pain-Related Outcomes in Patients on Long-Term Opioids

**DOI:** 10.2196/68292

**Published:** 2025-03-11

**Authors:** Karlyn A Edwards, Troy C Dildine, Dokyoung S You, Ashley M Herrick, Beth D Darnall, Sean C Mackey, Maisa S Ziadni

**Affiliations:** 1 Division of General Internal Medicine Department of Medicine University of Pittsburgh Pittsburgh, PA United States; 2 Division of Pain Medicine Department of Anesthesiology, Perioperative and Pain Medicine Stanford University Stanford, CA United States

**Keywords:** Empowered Relief, single session, chronic pain, prescription opioids, telehealth, daily data, pain intensity, pain catastrophizing

## Abstract

**Background:**

Patients with chronic pain on long-term opioid therapy often face barriers to accessing effective nonpharmacological treatments, including the burden of multiple sessions, lack of trained clinicians, and travel time. Empowered Relief (ER), a 2-hour, single-session pain relief skills class, can improve pain and quality of life among patients with chronic pain when delivered in person or virtually.

**Objective:**

This study examined the impact of Zoom-delivered ER among people with chronic pain on long-term opioid therapy. We assessed (1) the feasibility and acceptability of Zoom-delivered ER; (2) changes in pain and opioid use outcomes at 3 and 6 months after treatment; and (3) daily associations among pain, opioid dose, and the Pain Catastrophizing Scale (PCS) before and after treatment.

**Methods:**

During the early COVID-19 pandemic, we conducted an uncontrolled pilot study of a Zoom-delivered ER among 60 adults (n=45, 76% female participants; n=52, 88% White participants) experiencing chronic pain who were receiving daily prescribed opioids (≥10 morphine-equivalent daily dose). Participants completed assessments at enrollment, before class, after class, 3 months after treatment, and 6 months after treatment. Furthermore, participants completed 2 daily assessment periods (spanning 14 consecutive days) before and after the class. We used a multilevel modeling approach to examine (1) the raw changes in PCS, average pain intensity, pain interference, and self-reported opioid dose at 3 and 6 months after treatment and (2) daily-level changes in average pain intensity and opioid dose before and after the class.

**Results:**

Of the 60 participants enrolled, 41 (68%) attended the class and 24 (59% of the 41 class attendees) reported satisfaction with the Zoom-delivered class. PCS score was significantly reduced at 3 months (β=–3.49, *P*=.01; Cohen *d*=0.35) and 6 months after treatment (β=–3.61, *P*=.01; Cohen *d*=0.37), and pain intensity was significantly reduced at 3 months (β=–0.56, *P*=.01; Cohen *d*=0.39) compared to enrollment. There were no significant reductions in pain interference or opioid dose. Across daily assessments, higher daily pain catastrophizing was associated with worse daily pain (β=.42, *P*<.001) and higher self-reported opioid use (β=3.14, *P*<.001); daily pain intensity significantly reduced after the class (β=–.50, *P*<.001). People taking prescribed opioids as needed trended toward decreasing their daily opioid use after the class (β=–9.31, *P*=.02), although this result did not survive correction for multiplicity.

**Conclusions:**

Improvements to future Zoom-delivered ER iterations are needed to improve feasibility and acceptability among people with chronic pain and daily prescribed opioid use. Despite this, findings show a promising preliminary impact of the intervention on pain outcomes. A larger randomized controlled trial of Zoom-delivered ER among this patient population is currently under way.

## Introduction

### Background

One in 5 Americans has chronic pain [1], a condition associated with substantial personal and societal costs. Annual expenditures related to chronic pain, including lost productivity, exceed half a trillion dollars [2]. Individuals with chronic pain frequently report impacts on physical, psychological, and social functioning [3]. Over 100,000 Americans receive daily prescribed opioids [4], and while they can be an effective pain treatment for some [5], this treatment approach can also present several health risks [6], including overdose and death [7]. Furthermore, prolonged opioid use can contribute to mental health challenges, such as depression [8]. Thus, the gold standard for chronic pain management is a multimodal approach that integrates medical, behavioral, and rehabilitative treatments to minimize opioid use and maximize patient functioning [9,10].

Behavioral pain treatments are low-risk approaches that may reduce opioid-related risks and mitigate the broader impacts of chronic pain [11]. For example, cognitive behavioral therapy (CBT) alone [12], and when combined with posttreatment therapeutic interactive voice response telephonic support [13], has been shown to reduce opioid misuse. Moreover, acceptance and commitment therapy was shown to aid in opioid cessation [14] in patients flagged as being at risk for problematic opioid use after surgery. In addition, acceptance and commitment therapy was shown to aid in opioid reduction combined with mindfulness-based relapse prevention [15] in patients with prescription opioid misuse. In addition, a technique named Mindfulness-Orientated Recovery Enhancement (MORE) can lower pain interference and severity, as well as opioid misuse among people with comorbid chronic pain and opioid misuse [16]. Few interventions have been tested among patients who do not specifically misuse opioids, and the findings are mixed [13,17]. While studies have demonstrated the effectiveness of behavioral interventions, few patients can access them due to low availability and high treatment time burden (typically requiring 8-16 hours of in-person or online treatment) [18]. Poor insurance coverage and availability are primary barriers [19], in addition to travel time and the length of the interventions [20]. Treatment barriers were exacerbated by the COVID-19 pandemic, which caused further delays that contributed to worsened pain [21], function, and psychological distress [22,23]. Furthermore, treatment burdens limit both the accessibility and scalability of behavioral pain interventions, and in part, opioid prescribing may be the result of fewer available alternatives, particularly for patients in low-resource settings [24].

A single-session behavioral pain relief skills class, Empowered Relief (ER), was developed to address these barriers. ER synthesizes pain neuroscience education, cognitive behavioral skills, and mindfulness-based skills into a 2-hour, single-session pain relief skills class that enhances self-regulation of pain, including pain catastrophizing [25]. Pain catastrophizing, defined as a pattern of thinking that involves rumination, magnification of pain, and helplessness, is a robust predictor of worse pain and opioid use outcomes [26,27], including greater opioid use [28], opioid misuse [29], pain-related disability [30], and negative affect [31]. Previous work has demonstrated the efficacy of ER when delivered in person [32] and in synchronous [33] and asynchronous [34,35] web-based formats. Notably, when delivered via Zoom to patients with mixed chronic pain conditions (*not taking opioids*; N=101), it was superior to a waitlist control in reducing pain catastrophizing, pain intensity, and pain interference at 3 months after treatment [33]. A larger comparative efficacy trial in people with chronic low back pain (N=263) found in-person ER to be noninferior to 8 sessions of CBT for reducing pain catastrophizing, pain intensity, and pain interference (2 hours vs 16 hours of treatment time) at 3 [32] and 6 months [36]. In addition, 2 randomized controlled trials (RCTs) examining postsurgical outcomes showed that a web-based, automated version of ER tailored to surgical patients had opioid-sparing effects [34] and reduced pain [35] up to 3 months after surgery.

Critical gaps remain in disseminating high-quality digital pain treatments and using digital data collection methods, which can reduce barriers to participation in treatment and research studies [37,38]. Few studies have used digital daily assessments among people with chronic pain taking daily prescribed opioids [31,39-43], despite support for their feasibility and acceptability for measuring daily opioid use among patients with chronic pain [44]. Of those few studies, results consistently show heightened daily-level associations among pain catastrophizing, pain, and opioid craving and use [39,41-43]. However, only 2 studies have examined these associations before and after a behavioral pain intervention among people with chronic pain on long-term opioid therapy. Both studies found significant reductions in daily reports of pain, stress, and craving during and 1 month after a mindfulness-based intervention (MORE) [45,46] involving 16 hours of treatment. Further research of briefer behavioral pain interventions and their impact on daily-level pain and opioid use is needed.

### Objectives

We aimed to address these gaps by conducting an uncontrolled pilot study to assess the feasibility, acceptability, and impact of Zoom-delivered ER on pain and opioid use outcomes among patients with chronic pain receiving daily prescribed opioids. We hypothesized that at least 80% of enrolled patients would engage in the treatment. In addition, we hypothesized that ≥80% of patients would rate treatment acceptability, satisfaction, usefulness, and ease of understanding at ≥8 out of 10 and rate ease, comfort, and overall satisfaction of using the Zoom platform to be ≥5 out of 7 (higher scores indicating greater satisfaction, comfort, and ease). Second, we examined changes in pain and opioid use outcomes from enrollment to 3 months (primary end point) and 6 months after treatment. We hypothesized that there would be a significant reduction in pain intensity, pain interference, and pain catastrophizing at 3 and 6 months after treatment compared to enrollment. We examined changes in opioid dose as an exploratory outcome. Finally, we used 2 rounds of digital daily assessments before and after the class to characterize changes in daily associations between pain catastrophizing, pain intensity, and opioid use before and after the ER class. We hypothesized that higher daily pain catastrophizing would be significantly associated with higher pain intensity and self-reported opioid use. We also hypothesized that daily use of behavioral skills would be associated with a reduction in pain intensity and opioid use after class.

## Methods

### Study Design

The Stanford University School of Medicine coordinated this uncontrolled, prospective trial during the COVID-19 pandemic. We designed this study to assess the feasibility and acceptability of delivering ER via Zoom (Zoom Communications) to inform the transitioning of a larger RCT (ClinicalTrials.gov: NCT03950791) to the Zoom platform [47]. Thus, this study was not powered to detect differences in outcomes; however, findings will be used to determine adequate power and sample size for future studies. Reporting of the current trial is in concordance with the CONSORT (Consolidated Standards of Reporting Trials) 2010 extension for randomized pilot and feasibility trials. Participants with mixed-etiology chronic pain who were taking daily prescribed opioid medication and were between the ages of 18 and 80 years were enrolled. Full data collection, including all posttreatment assessments, was conducted between June 2020 and June 2021. The 2 ER classes occurred between June 2020 and November 2020.

### Ethical Considerations

This study protocol was submitted as a protocol modification to the larger clinical trial and was approved by the Stanford University Institutional Review Board (48784). To enroll in the study, electronic informed consent was obtained from all participants in concordance with the institutional review board protocol. Study data were deidentified and anonymized after study completion. Participants were compensated US $135 for completing the baseline, preclass, postclass, 3-month, and 6-month assessments. The class was provided at no charge. Participants who completed at least 10 of the 14 daily surveys were entered into a raffle for an iPad. Participants could be entered up to twice if they met this threshold for both the baseline and follow-up daily surveys.

### Recruitment and Enrollment

Participants were recruited remotely through targeted emails to lists of patients who agreed to be contacted for research purposes through the following three sources: (1) Stanford’s Learning Health System, comprising patients who have received care at the Stanford Pain Management Center (a referral-based tertiary outpatient pain clinic in Redwood City, California, that offers multidisciplinary pain care); (2) the Stanford Systems Neuroscience and Pain Lab database of community members with chronic pain who expressed interest in participating in research; and (3) study advertisements on the Stanford Pain Division website. Approximately 17% (10/60) of the study sample were clinic patients and 83% (50/60) were from the larger community. Patients who were in 1 of the 2 databases (Stanford’s Learning Health System or Stanford Systems Neuroscience and Pain Lab) were sent an email asking their interest in participating in a chronic pain treatment research study, and if interested, were directed to click on a link to a web-based screening form. Community members who saw the study advertisement on web were also directed to click on a link to fill out a web-based screening form. Those who met the initial eligibility criteria were contacted by research staff by phone to confirm their eligibility. Inclusion criteria were (1) individuals aged between 18 and 80 years; (2) individuals with noncancer chronic pain (defined as pain duration ≥6 months); (3) individuals who self-reported current use of prescription opioids ≥10 morphine-equivalent daily dose (MEDD) for ≥3 months; (4) individuals with fluency in the English language; and (5) individuals who were able to attend a one-time web-based class and complete web-based surveys. Exclusion criteria were (1) ongoing legal action or disability claim, (2) active participation in a CBT-based treatment, (3) inability to provide informed consent and complete study procedures, and (4) active suicidality. Eligible participants were asked to complete an electronic informed consent form and were immediately scheduled for the next available class and directed to fill out the web-based baseline assessment. Recruitment occurred in 2 waves, with recruitment windows open for the 6 weeks preceding each scheduled class.

### Assessments

All surveys were collected through REDCap (Research Electronic Data Capture; Vanderbilt University) [48], a web-based electronic data capture platform, which is a secure (password-protected), Health Insurance Portability and Accountability Act–compliant platform hosted by the Stanford University School of Medicine. Survey links were sent via SMS text message or email to participants. Participants completed their first survey at enrollment, followed by baseline daily surveys (14 consecutive days of daily surveys), which were initiated 2 to 4 weeks before the scheduled class. Three days before the class, participants completed a preclass survey, and immediately following the class, participants completed a postclass satisfaction survey. The follow-up daily surveys (14 consecutive days) were initiated 1 month after the class. All daily assessments were deployed at the same time each evening. Furthermore participants completed posttreatment surveys at 3 and 6 months after treatment (Multimedia Appendix 1 illustrates the study flow). There was no in-person contact with a study therapist or research staff. SMS text messages and phone call reminders were deployed by a research assistant to encourage completion of all assessments.

### Study Intervention: ER

ER was developed in 2013 [25] and compresses pain neuroscience education and key elements and skills from CBT-based pain and mindfulness interventions into a 2-hour single-session class [32,33]. Each class was led by 1 trained doctoral-level clinical psychologist using a PowerPoint (Microsoft Corporation) slide deck via Zoom to participant cohorts. Didactic content included psychoeducation about mind-body science as it relates to pain and negative mindset, as well as opioid safety. Participants learned skills on how to identify a negative mindset and self-treat it. During the class, participants practiced skills to decrease physiological hyperarousal, improved regulation of cognition and emotion, and engaged in adaptive behaviors to modulate attention and counteract helplessness. Skills included relaxation, cognitive reframing and thought restructuring, and identifying pleasant activities. Participants self-tailored the information described during the class and developed their own comprehensive self-treatment plan to stop and prevent catastrophizing. Participants left the class with a self-written personalized plan and a 20-minute binaural relaxation response app and were provided verbal and written instructions on how to download the app onto their mobile phone or portable electronic device.

### Class Platform

One week before the class, patients were emailed a Zoom link to join the class, along with information on when and how to attend the class. In addition, participants were offered a one-on-one Zoom training session with a research assistant before the intervention, if needed. Participant cohorts consisted of 19 to 22 participants per class. The Zoom platform was used to deliver the ER classes, and instructors screen-shared their PowerPoint presentation slides throughout the class. Class participants were encouraged to ask questions at any time through the Zoom chat box and unmute themselves to participate. Zoom classes were password-protected and hosted within the firewalled Stanford University School of Medicine and Stanford Healthcare systems.

### Training and Monitoring of Instructors

Instructors were doctoral-level clinical psychologists trained and certified in ER delivery before administering treatment. Existing treatment manuals and highly structured and standardized class content assured treatment fidelity. A research coordinator served as a fidelity rater and directly observed all classes. Cohort effects were minimized due to the highly structured single-session format and limited participant interaction.

### Measures

#### Feasibility and Acceptability

The assessment of the feasibility and acceptability of the ER Zoom-delivered intervention replicated published methods and was assessed immediately after the class [33]. We assessed feasibility by the proportion of participants that attended the class. Acceptability was assessed by participant ratings of treatment satisfaction, relevance, usefulness of information presented, ease of understanding, and likelihood to use the skills learned. Responses were measured on an 11-point Likert scale from 0 to 10, with higher scores indicating higher acceptability and satisfaction across respective items.

In addition, we assessed satisfaction with using the Zoom platform, such as ease of operating Zoom, engagement with the class material during the Zoom class, and ability to find a private location. We assessed comfort with the Zoom instructor and other class participants, connectedness to the Zoom instructor, as well as overall satisfaction with the Zoom platform. Ratings were assessed using a 7-point Likert scale from 1 “strongly disagree” to 7 “strongly agree,” with higher scores indicating greater satisfaction. Finally, we asked participants for open text responses regarding general feedback on the class and what they found helpful or challenging.

#### Pain and Opioid Use Measures

The following measures were collected at enrollment, before class (3 days before the class), and 3 and 6 months after treatment.

##### Pain Intensity

Respondents rated their average pain intensity over the previous 7 days on a numerical pain rating scale of 0 “no pain” to 10 “worst pain imaginable” [49,50].

##### Patient Reported Outcome Measurement Information System Pain Interference

PROMIS (Patient Reported Outcome Measurement Information System) pain interference short form (8 item) [51,52] was used to assess self-reported consequences of pain, including engagement with daily activities. Higher scores indicate higher interference with activities. The National Institutes of Health PROMIS short-form measures have been applied in pain research [51], and selected domains were identified by the Initiative on Methods, Measurement, and Pain Assessment in Clinical Trials as core outcomes [53]. The web-based PROMIS assessment center software [54] was used to calculate the short-form T scores. A standardized T score for each PROMIS measure is generated for each patient. A score of 50 reflects the mean of the US general population, with an SD of 10. Internal consistency of the pain interference measure at baseline was high (α=.96).

##### Pain Catastrophizing Scale

The 13-item Pain Catastrophizing Scale (PCS) [26] was used to measure negative thoughts and emotional responses to pain. PCS includes 3 subscales: helplessness, magnification, and rumination. The response scale ranges from 0 “not at all” to 4 “all the time”; total sum scores range from 0 to 52. It is scored by summing all items, with higher scores indicating greater catastrophizing. The PCS has demonstrated validity and reliability in mixed-etiology chronic pain and is a psychometrically trusted instrument [55]. The internal consistency of the PCS in our sample at baseline was high (α=.93).

##### Opioid Dose

###### Overview

Self-reported opioid type and dose was converted to MEDD using the US Department of Health & Human Services Opioid Oral Morphine Milligram Equivalent Conversion Factors table [56] and the State of Ohio Board of Pharmacy Oral Morphine Milligram Equivalent Conversion Table [57]. Our methods reflected self-reported daily opioid use captured in other trials [34,35,58]. A board-certified anesthesiology and pain medicine physician verified the calculations. In addition, patients were categorized into one of four opioid cohorts based on their opioid prescription type at enrollment: (1) short-acting prescribed opioids as needed (pro re nata; PRN); (2) prescribed consistent, long-acting opioids; (3) prescribed buprenorphine; and (4) intrathecal pump.

The following measures were collected during the baseline (2-4 weeks before the class) and follow-up (1 month after the class) daily survey periods. Daily behavioral skill use was only assessed during the follow-up daily survey period.

##### Daily PCS

The daily PCS is a 3-item version of the PCS developed for use in daily diary studies to facilitate research on mechanisms of catastrophizing treatment. It has demonstrated validity and reliability in patients with chronic pain [59]. Scores were calculated by summing all items with a range from 0 to 12. Items assessed patterns of unhelpful thoughts about pain within the last 24 hours and were rated on a 5-point Likert scale from 0 “not at all” to 4 “all the time.” Example items included, “During the past 24 hours I kept thinking about how much I hurt.” The internal consistency of the follow-up daily PCS in our sample was high (α=.91).

##### Daily Pain Intensity

Daily average pain ratings were assessed on a 0 to 10 numerical rating scale (0=no pain and 10=worst pain imaginable) based on pain experienced in the past 24 hours [49,60].

##### Daily Opioid Dose

Daily opioid dose, as reported by participants, was converted to MEDD using the US Department of Health & Human Services Opioid Oral Morphine Milligram Equivalent Conversion Factors table [56] and the State of Ohio Board of Pharmacy Oral Morphine Milligram Equivalent Conversion Table [57]. Our methods reflected self-reported daily opioid use captured in other trials [34,35,58]. A board-certified anesthesiology and pain medicine physician verified the calculations.

##### Daily Skills Use

Skills use was assessed for 14 days during the follow-up daily assessments only. Three items were asked to assess the frequency of cognitive, behavioral, and relaxation techniques used over the past 24 hours using a 6-point Likert scale ranging from 0 “No use” to 5 “Five or more times.” The items were “How many times did you engage in deep breathing or relaxation today?” “How many times did you use distraction or reframing today?” and “How many times did you use pain relief actions today?”

#### Data Analysis Plan

Analyses were carried out using SPSS (IBM Corp) [61] and RStudio (RStudio, Inc) [62], a per-protocol approach was used. Descriptive data were calculated, including means and SDs across all study variables and time points. To examine attrition, differences in baseline demographic, pain, and opioid characteristics were assessed between those with complete and missing data at each study time point using chi-square tests and ANOVAs. To examine treatment satisfaction, average participant ratings across each satisfaction item were calculated, as well as the proportion of participants who rated each item ≥8 out of 10. To examine satisfaction with the Zoom platform, average participant ratings across each satisfaction item were calculated as well as the proportion of participants who rated each item strongly agree, agree, or somewhat agree (≥5 out of 7).

To examine raw changes in pain intensity, pain catastrophizing, pain interference, and opioid dose from enrollment to 3 and 6 months after treatment, multilevel models with a subject-level random intercept and a fixed effect of time were used. In total, 4 time points (enrollment, before class, 3 months, and 6 months) were included in each model. Linear multilevel models were used to assess changes in pain intensity, pain interference, and pain catastrophizing, and a negative binomial multilevel model was used to assess changes in opioid dose (MEDD), given the highly skewed distribution. Negative binomial models use a natural logarithm link function to account for overdispersion and skewed data. The raw coefficients from this model were converted to a log scale and interpreted as incidence rate ratios, which describe the proportional change in the outcome associated with a 1-unit increase of the predictor [63]. Furthermore, we ran sensitivity analyses for each model, removing tramadol, buprenorphine, and methadone users from the analysis (due to difficulty in equating MEDD). Finally, we calculated Cohen *d* effect sizes (0.20=small, 0.50=medium, and 0.80=large) [64] to aid interpretation.

Across the daily assessments, a similar multilevel modeling approach was used to take advantage of the repeated-measures design. All multilevel models included random intercepts at the participant level and fixed effects of each predictor. To examine predictors of change in daily average pain intensity across the baseline and follow-up daily assessments (relative to enrollment), a linear multilevel model was used. Predictors included daily pain catastrophizing; time (dichotomized factor of pre- vs postclass assessment); dummy-coded opioid cohort (PRN, long-acting, buprenorphine, and intrathecal pump cohorts); and time’s interactions with each factor. Change in opioid dose (MEDD) was transformed into percentage change to approximate a normal distribution. Outliers at the upper bound were windsorized [65] to the 95th percentile (Multimedia Appendix 2 provides more details). A linear multilevel model was used to examine predictors of percentage change in opioid dose across the baseline and follow-up daily assessments (relative to enrollment). Predictors included daily pain catastrophizing, daily average pain intensity, time (before vs after class), opioid cohort, and time’s interaction with each factor. For both the multilevel models assessing the time and daily predictors, we ran sensitivity analyses for each model, removing tramadol and methadone users from the analysis (due to difficulty in equating MEDD).

Finally, to examine the role of behavioral skill use on changes in pain and opioid dose, 2 additional linear multilevel models were conducted across the follow-up daily assessments. Each behavioral skill was dichotomized into no use and ≥1 use in the past 24 hours and added as predictors in each model. Only dummy-coded PRN and long-acting opioid cohorts were examined due to small sample sizes within intrathecal pump and buprenorphine cohorts and rank deficiency errors with maximal models. To examine changes in average pain intensity, predictors included daily pain catastrophizing, time (before vs after class), PRN versus long-acting cohort, behavioral skills (relax, reframe, and actions), and time’s interaction with every factor. To examine the percentage change in opioid dose, predictors included daily pain catastrophizing, daily average pain intensity, time (before vs after class), PRN versus long-acting cohort, behavioral skills (relax, reframe, and actions), and time’s interaction with every factor.

Multimedia Appendix 2 gives a description of pain and opioid dose outcome calculations and all model specifications. Across all models, data were inspected to ensure model assumptions were met (ie, homoscedasticity in residuals), and *P* values were corrected for multiple comparisons using the Benjamini-Hochberg false discovery rate (BH-FDR) procedure, an alternative measurement to the family-wise error rate [66]. The BH-FDR procedure has been shown to have greater power than classical family-wise error rate procedures (eg, Bonferroni), while still adequately controlling for type 1 errors. The desired α level (α=.05), number of tests, and raw *P* value for each test were used to calculate the BH-FDR [66].

## Results

### Overview

Participants were primarily female (45/59, 76%), White (52/59, 88%), non-Hispanic or Latino (57/59, 97%), married (28/59, 48%), and not working due to pain (28/59, 47%). The most prevalent pain condition was chronic low back pain (35/59, 58%); most had pain >5 years (54/59, 92%; Table 1). Oxycodone was the most commonly prescribed opioid (14/59, 24%; Multimedia Appendix 3 for all opioid types). The use of PRN opioids was most common among the sample (32/59, 78%), followed by long-acting prescribed opioids taken consistently (16/59, 39%), buprenorphine (10/59, 24%), and intrathecal pump (2/59, 5%). Table 2 provides descriptive data for all monthly and daily-level variables.

**Table 1:** The enrolled sample demographic characteristics of people with chronic pain prescribed daily opioids^a^.

Measure			Values
Age (y), mean (SD)			59.13 (14.54)
**Gender, n (%)**			
	Men	13 (22)	
	Women	45 (76)	
	Other	1 (2)	
**Race, n (%)**			
	American Indian or Alaska Native	2 (3)	
	Asian	1 (2)	
	Black or African American	2 (3)	
	White	52 (87)	
	>1 race	1 (2)	
	Other	1 (2)	
**Ethnicity, n (%)**			
	Hispanic or Latino	2 (3)	
	Non-Hispanic or Latino	57 (95)	
**Marital status, n (%)**			
	Never married	8 (13)	
	Partnered	8 (13)	
	Married	28 (47)	
	Separated	1 (2)	
	Widowed	4 (7)	
	Divorced	10 (17)	
**Education, n (%)**			
	Some high school	1 (2)	
	High school diploma or GED^b^	1 (2)	
	Some college, no degree	17 (28)	
	Associates or vocational degree	10 (17)	
	Bachelor’s degree	18 (30)	
	Master’s, professional, or doctoral degree	12 (20)	
**Annual household income (US $), n (%)**			
	<24,999	19 (32)	
	25,000-64,999	9 (15)	
	65,000-84,999	6 (10)	
	85,000-124,999	11 (18)	
	>125,000	14 (23)	
**Employment^c^, n (%)**			
	Full time or part time	9 (15)	
	Homemaker or not employed	5 (8)	
	Not working due to pain	28 (47)	
	Retired	20 (33)	
**Receiving disability, n (%)**			
	Yes	19 (32)	
	No	40 (67)	
**Chronic pain condition^c^, n (%)**			
	Postsurgical recovery	13 (22)	
	Chronic low back pain	35 (58)	
	Complex regional pain syndrome	7 (12)	
	Pelvic pain	7 (12)	
	Migraines	15 (25)	
	Headaches	9 (15)	
	Neuropathic pain	33 (55)	
	Abdominal pain	11 (18)	
	Myofascial pain	23 (38)	
	Fibromyalgia	14 (23)	
	Other	22 (37)	
**Pain duration (y), n (%)**			
	1-5	5 (8)	
	>5	54 (90)	

^a^In total, 60 participants enrolled but 1 withdrew and did not provide demographic information.

^b^GED: General Educational Development.

^c^The participants could select all that apply.

**Table 2:** Descriptive data of monthly and daily-level study variables across all study time points (enrollment through 6 months) among people with chronic pain prescribed daily opioids^a^.

	Enrollment	Baseline daily dairies	Before class	Follow-up daily dairies	3 mo	6 mo
Total, n (%)	60 (100)	56 (93)	53 (88)	40 (67)	38 (63)	36 (60)
Pain intensity, mean (SD)	5.76 (1.71)	5.56 (2.10)	5.58 (1.70)	4.64 (2.30)	5.07 (1.80)	5.34 (2.03)
Pain catastrophizing, mean (SD)	17.29 (10.48)^b^	3.90 (2.90)^c^	15.30 (9.83)^b^	3.10 (2.92)^c^	13.32 (12.12)^b^	13.14 (11.61)^b^
Pain interference, mean (SD)^d^	63.49 (7.45)	—^e^	62.38 (7.28)	—	61.26 (9.10)	61.00 (10.38)
Opioid dose^f^, mean (SD)	92.11 (147.13)	96.01 (166.62)	102.84 (171.06)	74.10 (120.79)	84.03 (136.30)	87.42 (136.30)

^a^Daily dairies were completed for 14 days approximately 2 to 4 weeks before the class (baseline daily dairies) and for 14 days approximately 4 weeks after the class (follow-up daily dairies). Means and SDs for this period were calculated across all 14 days.

^b^13-item Pain Catastrophizing Scale.

^c^3-item daily Pain Catastrophizing Scale.

^d^Patient Reported Outcome Measurement Information System pain interference scale *t* score.

^e^Not available.

^f^Opioid dose as measured by morphine-equivalent daily dose.

A total of 60 participants enrolled in the study, and 7% (n=4) withdrew from the study before completing the baseline survey (7% pretreatment attrition). Of the 56 (N=60, 93%) participants who completed the baseline survey, 41 (73%) participants attended the class (68% of the enrolled sample), 38 (63% of the enrolled sample) completed the 3-month follow-up survey, and 36 (60% of the enrolled sample) completed the 6-month follow-up survey (Multimedia Appendix 1 illustrates the study flowchart). Attrition analyses indicated that those who did not attend the class or complete the follow-up daily surveys were more likely to be female participants (χ^2^_40_=8.72, *P*=.01). This pattern held at the 3-month posttreatment assessment (gender: χ^2^_37_=6.51, *P*=.04). No other differences were observed in study variables or demographic characteristics between those who had complete or missing data at any other study time points.

### Feasibility and Acceptability

Some (41/60, 68% of the enrolled sample) attended the class and completed the postclass survey. Of those who attended, 59% (24/41) of the sample rated Zoom-delivered ER to be satisfactory (ie, ≥8 out of 10; mean 8.02, SD 2.08), and 73% (30/41) rated the class easy to understand (mean 7.93, SD 2.1). Some (26/41, 63%) rated the class as useful (mean 7.83, SD 2.27), 63% (26/41) rated the class as relevant (mean 8.10, SD 2.21), and 77% (31/41) stated they were likely to use the skills (mean 8.41, SD 1.94). Open feedback was collected about the class, and 63% (26/41) provided comments. The most common feedback was that the class reduced travel barriers (15/41, 37%), increased comfort by being able to participate at home (13/41, 32%), and reduced concerns about the COVID-19 pandemic (6/41, 15%). Some (7/41, 17%) participants reported difficulty engaging in the relaxation exercise or the class due to pain or fatigue, 14% (6/41) reported a preference for an in-person class, and 12% (5/41) had been exposed to similar content in the past or were unsure if it would be helpful for their specific pain condition.

Nearly all participants (39/41, 95%) found the Zoom platform easy to operate (≥5 out of 7; mean 6.44, SD 0.92); 97% (40/41) easily found a private location to attend the class (mean 6.71, SD 0.68), and 93% (38/41) reported ease in engaging in the class material via Zoom (mean 6.05, SD 1.24). Some (26/41, 63%) participants reported feeling connected to the instructor (mean 5.22, SD 1.64). Overall, 95% (39/41) of participants reported being somewhat satisfied (n=3, 7%), satisfied (n=17, 42%), or completely satisfied (n=19, 46%) with the Zoom platform (mean 6.24, SD 0.97). A majority (33/41, 80%) of participants reported no problems with the Zoom platform. Of those who reported a problem (n=8, 20%), the most frequently cited problem was unstable internet connection and poor audio quality (n=5, 12%).

### Pain and Opioid Use Outcomes

Findings from the 4 multilevel models examining the effect of time on raw changes in pain and opioid outcomes are outlined in Table 3. The corrected significance thresholds for each model were pain intensity (*P*=.008), pain interference (*P*=.001), pain catastrophizing (*P*=.01), and opioid dose (*P*=.05). Results showed a significant reduction in pain catastrophizing from enrollment to 3 months (mean –2.89, SD 10.89), and from enrollment to 6 months (mean –2.83, SD 10.89) both with a small effect size (Cohen *d*=0.35 and 0.37, respectively). The average pain intensity significantly reduced from enrollment to 3 months posttreatment (mean –0.52, SD 1.40), indicating a small effect size (Cohen *d*=0.39). However, average pain intensity did not maintain a significant change from enrollment to 3 months in our sensitivity analysis (Multimedia Appendix 4) nor from enrollment to 6 months posttreatment (mean –0.24, SD 1.42). Reductions in pain interference from enrollment to 3 months (mean –2.03, SD 4.94) and 6 months (mean –2.14, SD 6.77) did not survive correction, although they demonstrated small effect sizes (Cohen *d*=0.27 and 0.28, respectively). There were no significant reductions in opioid dose across the monthly study time points.

**Table 3:** Multilevel regressions examining changes in pain and opioid use outcomes from baseline through 6-month time points among people with chronic pain prescribed daily opioids.

Outcomes and variables			Coefficient^a^ (SE)	Test statistic^a^	*P* value	Cohen *d*^b^
**Linear**						
	**Pain intensity**					
		Intercept	5.76 (0.23)	24.68	<.001^c^	—^d^
		Before class^e^	–0.16 (0.19)	–0.84	.40	0.10
		3 mo^e^	–0.56 (0.21)	–2.67	.008^c^	0.39
		6 mo^e^	–0.29 (0.21)	–1.37	.17	0.22
	**Pain interference**					
		Intercept	63.49 (1.09)	58.02	<.001^c^	—
		Before class^e^	–1.06 (0.77)	–1.36	.18	0.15
		3 mo^e^	–1.98 (0.88)	–2.25	.03	0.27
		6 mo^e^	–1.95 (0.89)	–2.18	.03	0.28
	**Pain** **c** **atastrophizing**					
		Intercept	17.29 (1.44)	12.05	<.001^c^	—
		Before class^e^	–2.07 (1.22)	–1.70	.09	.20
		3 mo^e^	–3.49 (1.38)	–2.53	.01^c^	.35
		6 mo^e^	–3.61 (1.40)	–2.57	.01^c^	.37
**Negative binomial**						
	**Opioid dose (MEDD^f^)**					
		Intercept	46.50 (7.13)	25.00	<.001^c^	—
		Before class^e^	1.02 (0.07)	0.29	.77	–0.07
		3 mo^e^	0.89 (0.07)	–1.52	.13	0.06
		6 mo^e^	0.96 (0.07)	–.48	.63	0.03

^a^The linear multilevel model reports an unstandardizedβ coefficient and *t* value statistics. The negative binomial multilevel model, via glmer, reports incidence rate ratios and *z*-value statistics.

^b^The effect size *d* calculated as baseline minus follow-up divided by pooled SD. Positive *d* values indicate improvements.

^c^Significant values based on the corrected *P* value.

^d^Not available.

^e^Reference group is the baseline.

^f^MEDD: morphine-equivalent daily dose.

**Figure 1:**
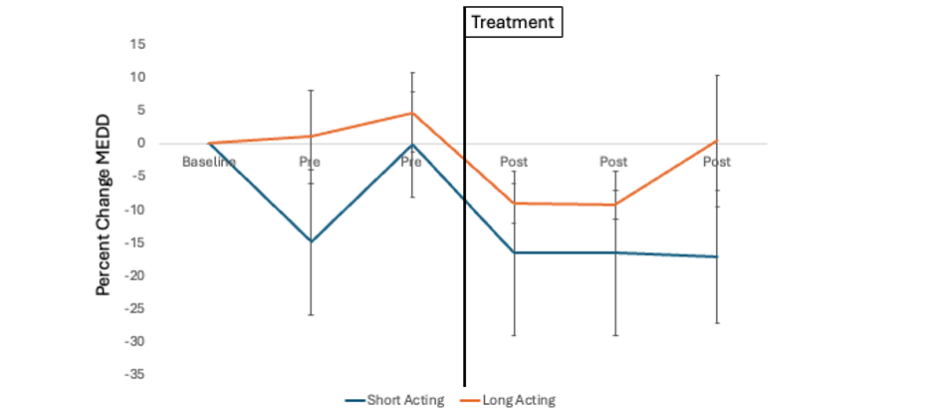
The raw percentage change in opioid dose (morphine-equivalent daily dose; MEDD) from enrollment across study time points between participants with chronic pain taking long-acting opioids and short-acting (prescribed opioids as needed) opioids.

Across the 2-week baseline daily assessment period (before class), 93% (56/60) of participants completed 95.5% (749/784) of deployed daily surveys. Across the 2-week follow-up daily assessment period (after class), 68% (41/60) of participants completed 95.6% (549/574) of deployed daily surveys. From the 41 participants who attended class, 1136 baseline and follow-up daily surveys were used to estimate changes in pain and opioid dose using linear multilevel models (Table 4). The corrected significance thresholds for each model were pain intensity (*P*=.001) and opioid dose (*P*=.003). Results showed that higher daily pain catastrophizing was associated with increases in average pain intensity across the daily assessments relative to enrollment (β=0.42, SE 0.02, *P*<.001), and there were greater reductions in pain across the follow-up daily surveys (after class) compared to the baseline daily surveys (before class; β=–0.51, SE 0.12, *P*<.001) although this effect did not survive sensitivity analyses (β*=*–0.41, SE 0.13*, P=*.002; Multimedia Appendix 5). Opioid cohort and all interaction factors were not significantly associated with change in average pain intensity. When examining changes in opioid dosage, higher daily pain catastrophizing (β=3.14, SE 0.74, *P*<.001) and daily average pain (β=3.47, SE 1.16, *P*=.003) were associated with increases in opioid dose across the daily assessments relative to enrollment, although the effect of pain did not survive our sensitivity analyses (β=3.58, SE 1.40, *P*=.01). Those taking short-acting opioids as needed (PRN) showed a trend toward reduced opioid dose across the daily assessments (β=–29.88, SE 10.68, *P*=.03), although this association did not survive correction. Furthermore, the time × PRN cohort interaction did not survive correction, although it indicated a trend toward greater opioid dose reductions for the PRN cohort across the follow-up daily surveys (after class) compared to the baseline daily surveys (before class; β=–9.31, SE 3.94, *P*=.02). Figure 1 depicts the raw percentage change in opioid dose between the long-acting and PRN cohorts.

**Table 4:** Multilevel linear regressions predicting change in pain intensity and opioid dose from enrollment across the baseline (before class) and follow-up (after class) daily diaries among people with chronic pain prescribed daily opioids.

Outcome and variables		Coefficient^a^ (SE)	Test statistic^a^	*P* value
**Change in pain intensity^b^**				
	Intercept	–0.42 (0.46)	–0.92	.36
	PCS^c,d^	0.42 (0.02)	18.94	<.001^e^
	Pre-post^f^	–0.51 (0.12)	–4.20	<.001^e^
	PRN^g^ cohort	–0.55 (0.58)	–0.96	.34
	Long-acting cohort	0.23 (0.65)	0.36	.72
	Buprenorphine cohort	–0.82 (0.69)	–1.18	.25
	ITP^h^ cohort	0.77 (1.45)	0.53	.60
	Pre-post x PCS	0.03 (0.03)	0.99	.32
	Pre-post x PRN	0.21 (0.15)	1.39	.16
	Pre-post x LA^i^	–0.27 (0.17)	–1.58	.12
	Pre-post x Buprenorphine	–0.04 (0.18)	–0.20	.84
	Pre-post x ITP	–0.32 (0.39)	–0.82	.41
**Percentage change in opioid dose^j^**				
	Intercept	5.77 (9.66)	0.60	.55
	PCS^d^	3.14 (0.74)	4.23	<.001^e^
	Pre-post^e^	1.40 (3.11)	0.45	.65
	Average pain	3.47 (1.16)	2.99	.003^e^
	PRN cohort	–26.88 (12.21)	–2.20	.03
	LA cohort	–17.36 (13.65)	–1.27	.21
	Buprenorphine cohort	–6.97 (14.59)	–0.48	.64
	ITP cohort	–17.64 (30.51)	–0.58	.56
	Pre-post × PCS	–1.72 (1.13)	–1.52	.13
	Pre-post × pain	–1.55 (1.78)	–0.87	.38
	Pre-post × PRN	–9.31 (3.94)	–2.37	.02
	Pre-post × LA	3.08 (4.39)	0.70	.48
	Pre-post × buprenorphine	–8.93 (4.67)	–1.92	.06
	Pre-post × ITP	16.98 (9.90)	1.72	.09

^a^The linear multilevel model reports an unstandardized β coefficient and *t* value statistics. ^b^The outcome is change in average pain intensity in the past 24 hours and depicts a change at each daily assessment compared to the enrollment.

^c^PCS: Pain Catastrophizing Scale.

^d^Daily PCS.

^e^Significant values based on the corrected *P* value.

^f^0: preclass daily assessments; 1: postclass daily assessments.

^g^PRN: pro re nata.

^h^ITP: intrathecal pump.

^i^LA: long acting.

^j^The percentage change in opioid dose is measured using morphine-equivalent daily dose and depicts a change at each daily assessment compared to the enrollment.

Across the follow-up daily surveys (1 month after the class; n=549), 446 (81.2%) of the daily surveys endorsed using relaxation at least once, 403 (73.4%) endorsed using reframe at least once, and 410 (74.7%) endorsed using pain relief actions at least once. Findings from the multilevel models are provided in Multimedia Appendix 6. The corrected significance thresholds for each model were pain intensity (*P*=.001) and opioid dose (*P*=.05). Results showed that higher daily pain catastrophizing was associated with increase in average pain intensity relative to enrollment (β=0.48, SE 0.04, *P*<.001). The use of behavioral skills, time, and interactions was not a significant predictor of change in average pain intensity. Furthermore, daily pain catastrophizing, daily pain, use of the behavioral skills, time, or their interactions were not significant predictors of percentage change in opioid dose across the follow-up daily surveys.

## Discussion

### Principal Findings

We performed an uncontrolled pilot study to investigate the initial feasibility, acceptability, and preliminary impact of a Zoom-delivered ER class among people with chronic pain taking daily opioid medication. Our results showed significant reductions in pain catastrophizing and average pain intensity that lasted 3 months after treatment, although with small effects. Pain catastrophizing, a target of the ER class, continued to exhibit significant reductions at 6 months after treatment. Daily-level results also showed a trend toward opioid dose reduction for people taking PRN opioids, though did not survive correction (*P*=.02). The findings from this pilot study provide justification for a larger, appropriately powered RCT.

### Feasibility and Acceptability

We conducted this study during the first year of the COVID-19 pandemic (2020 to 2021), while access to traditional, in-person pain psychology interventions was extremely limited. Although we observed lower-than-hypothesized engagement rates (≥80%), with 68% (41/60) of the enrolled patients attending the class, we noted that our engagement was relatively high when compared to the substantially reduced health care use that occurred during the early stages of the COVID-19 pandemic [21]. For those who participated in the class, 63% (26/41) to 77% (31/41) of the patients rated the class as satisfactory, useful, and easy to understand. Furthermore, they reported high satisfaction with web-based delivery, which reduced travel barriers and concerns about the COVID-19 pandemic and increased comfort by attending the intervention from home. In addition, ease and comfort with digital assessments was evident, as >93% (749/784) of the offered daily surveys were completed (ie, at least 26 of 28 days per person). Future work that involves follow-up interviews with participants is needed to identify potential adaptations to improve the intervention’s engagement and acceptability. On the basis of our initial findings, implementing strategies to address digital literacy and poor internet access may improve engagement, along with further tailoring of the relaxation exercise to better engage people with more severe pain and fatigue. In addition, increased reminders and incentives may increase engagement in the intervention.

### Pain and Opioid Use Outcomes

Following the ER class, individuals reported significant reductions in pain catastrophizing at 3 and 6 months after treatment and significant reductions in pain intensity at 3 months after treatment. Furthermore, results showed a trend toward decreased pain interference at 3 and 6 months after treatment, although these did not survive multiplicity correction (*P*=.03). This study of people taking daily opioids yielded smaller effect sizes (Cohen *d=*0.35-0.39) as compared to prior RCTs of in-person ER [32] (Cohen *d=*0.43-0.89) and Zoom-delivered ER [33] (Cohen *d=*0.54-0.76) in patients with chronic pain with no enrollment criterion applied for daily opioid use. While there are notable methodological differences between studies (eg, different pain conditions, enrollment criteria, and control conditions), the smaller effect sizes may also be due to higher rates of disability, which were 32% in this sample as compared to 5% to 10% in the previous 2 studies. Patients on long-term opioid therapy may require more intensive treatment, a different type of treatment, or booster sessions, especially in the setting of greater disability. Regardless, this study’s findings suggest that Zoom-delivered ER may impart small benefits in pain intensity and pain catastrophizing, and trending toward improvement for pain interference (though did not survive correction, *P*=.03), in people with chronic pain on long-term opioid therapy, warranting further testing in an RCT.

Our study is among the few to use daily digital assessments among patients with mixed-etiology chronic pain and taking long-term opioid medication. Our findings replicate previous work showing that higher daily reports of pain catastrophizing are associated with worse pain intensity, and higher daily pain intensity and pain catastrophizing are associated with taking greater doses of prescribed opioids [31,39,59]. In addition, daily ratings of pain decreased from pre-to-post class, similar to previous work that showed a mindfulness intervention (MORE) reduced daily-level pain, negative affect, opioid craving, and opioid use [45,46]. Furthermore, we examined the differences between patients prescribed PRN opioids and long-acting opioids. Results showed a trend that people prescribed PRN opioids were more likely to reduce their opioid dose from before to after class as compared to patients on long-acting opioids, although this association did not survive our rigorous multiple comparison correction (*P*=.02; Figure 1 illustrates a raw percentage change across both groups). Our findings provide a signal that a brief, Zoom-delivered behavioral pain treatment may help patients taking PRN opioids rely less on their medication, as has been found with other multisession behavioral treatments [15,16]. For those on long-acting opioids, patients are likely to need additional support and treatments, with active clinician-patient engagement targeting a slow, gradual weaning of their opioid dose over several weeks or months [67]. Behavioral treatments and psychoeducation are important treatment components as they offer patients additional pain coping skills and may help reduce potential pain flares during the weaning process [17,68,69]. Future RCTs are warranted to examine Zoom-delivered ER alone in reducing PRN opioid use and in the context of an opioid weaning protocol in conjunction with prescribers.

### Limitations

We noted several limitations in this study that are important to consider when interpreting the findings. First, this study was an uncontrolled pilot trial. Therefore, inferences about intervention causation cannot be assumed. We did use a rigorous statistical approach to control for potential type 1 errors. Furthermore, the actual therapeutic effect size cannot be ascertained and separated from potential regression to the mean effects. A larger RCT (NCT03950791) is currently underway to further assess the efficacy of Zoom-delivered ER among patients with chronic pain receiving daily prescribed opioids [58]. Second, our sample consisted of largely White individuals, non-Hispanic or Latino individuals, and educated women, although our sample did report higher rates of disability (19/59, 32%) than in our previous studies. Future RCTs are needed to assess the efficacy of the intervention in diverse and underserved patient populations who experience greater barriers to treatment [70]. Third, this study evidenced significant attrition (only 41/60, 60% of the enrolled sample completed the final 6-month assessment). While we did not find significant differences between those with complete versus missing data on our study variables, stressors related to the COVID-19 pandemic likely impacted study attrition. Importantly, we had exceptional compliance rates with digital daily assessments (93%-95%). Future research may consider using additional engagement strategies, such as increased digital assessments or phone calls in-between study time points. Fourth, we suspect that we did not see any significant associations between daily behavioral skill use and daily pain or opioid use because of the once-daily assessments that were completed at the same time each day and asked participants to recall information from the last 24 hours. Future research should use stratified random sampling, prompts with a shorter recall window, and other objective measures (eg, electronic pill caps) to reduce measurement error [71]. Finally, there are other variables that we did not measure that, in previous work, have moderated associations between pain and opioid use, such as opioid craving [72] and positive affect [45]. Future research using larger samples would benefit from modeling both known risk factors (eg, opioid craving, negative affect, and pain catastrophizing) [39,72] and protective factors (eg, positive affect and use of behavioral skills) [45] to further elucidate their impact on daily reports of pain and opioid use as well as their change following behavioral pain treatments.

### Conclusions

Improvements to future Zoom-delivered ER iterations are needed to improve feasibility and acceptability among people with chronic pain and daily prescribed opioid use. However, despite this, we also found a promising preliminary impact of the intervention on pain outcomes. There was high compliance with digital daily assessments, and results showed a promising trend of opioid dose reduction among people taking PRN opioids (though did not survive correction, *P*=.02). Our results indicate significant reductions in pain catastrophizing and daily pain intensity, suggesting that even a brief, digitally delivered behavioral intervention can positively influence pain outcomes in this population. Although reductions in opioid use did not reach statistical significance (*P*=.02), the observed trend among patients prescribed PRN opioids highlights ER’s potential role in supporting reduced reliance on medication.

We developed this study to inform a larger RCT that aims to rigorously assess the efficacy of Zoom-delivered ER using digital daily and monthly level assessments currently in data collection [58]. Our initial findings suggest that Zoom-delivered ER may be a promising intervention that can improve pain-related outcomes and reduce common barriers to accessing pain psychology interventions, such as travel and time burdens [20]. Dissemination of brief, accessible, and scalable pain psychology interventions, such as ER, stands to improve patient outcomes by increasing equitable access to effective pain psychology treatments and may be useful tools to help reduce pain and opioid use. ER offers a promising approach to expanding the reach of behavioral pain treatments, particularly for populations with limited access to in-person care.

## Appendix

Multimedia Appendix 1 Participant flowchart.

Multimedia Appendix 2 Supplementary methods.

Multimedia Appendix 3 Type of prescribed opioid used at enrollment.

Multimedia Appendix 4 A sensitivity analysis of multilevel regressions examining changes in pain and opioid outcomes across monthly assessments.

Multimedia Appendix 5 A sensitivity analysis of multilevel linear regressions predicting change in pain intensity and opioid dose from baseline across the baseline (before class) and follow-up (after class) daily dairies.

Multimedia Appendix 6 Multilevel linear regressions predicting change in pain intensity and opioid dose from enrollment across the follow-up (after class) daily dairies.

## Abbreviations

BH-FDR: Benjamini-Hochberg false discovery rate
CBT: cognitive behavioral therapy
CONSORT: Consolidated Standards of Reporting Trials
ER: Empowered Relief
MEDD: morphine-equivalent daily dose
MORE: Mindfulness-Orientated Recovery Enhancement
PCS: Pain Catastrophizing Scale
PRN: pro re nata
PROMIS: Patient Reported Outcome Measurement Information System
RCT: randomized controlled trial
REDCap: Research Electronic Data Capture

## Data Availability

The data generated are available upon reasonable request to the corresponding author (MSZ).
