# Supplementary material for: Zoom-Delivered Empowered Relief for Chronic Pain: Observational Longitudinal Pilot Study Exploring Feasibility and Pain-Related Outcomes in Patients on Long-Term Opioids
Source: JMIR Form Res. 2025 Mar 11;9:e68292. doi: 10.2196/68292 (PMC11937707; doi:10.2196/68292)
Supplement: Multimedia Appendix 1 [file formative_v9i1e68292_app1.docx]

**Multimedia Appendix 1**

Supplemental Figure 1. Participant flow chart.

Did not reach or declined participation (n = 24)

Contacted (N = 84)

Enrolled (N = 60)

Withdrew (n = 2; could not complete surveys)

Lost to follow up (n = 2)

Completed baseline survey (N = 56)

Lost to follow up (n = 2)

Completed baseline daily dairies (N = 54)

Lost to follow up (n = 1)

Completed pre-class survey (N = 53)

Lost to follow up (n = 12)

Attended class and completed post-class survey (N = 41)

Withdrew (n = 1; COVID-related stress)

Completed follow up daily dairies (N = 40)

Lost to follow up (n = 2)

Completed 3-month survey (N = 38)

Lost to follow up (n = 2)

Completed 6-month survey (N = 36)
