# Supplementary material for: Zoom-Delivered Empowered Relief for Chronic Pain: Observational Longitudinal Pilot Study Exploring Feasibility and Pain-Related Outcomes in Patients on Long-Term Opioids
Source: JMIR Form Res. 2025 Mar 11;9:e68292. doi: 10.2196/68292 (PMC11937707; doi:10.2196/68292)
Supplement: Multimedia Appendix 2 [file formative_v9i1e68292_app2.docx]

**Multimedia Appendix 2**

Supplementary Methods

*Data Analysis Plan*

To examine raw changes in pain and opioid dose outcomes, three linear multi-level regressions (predicting average pain intensity, pain catastrophizing, and pain interference) and one negative binomial multi-level regression (predicting Morphine Equivalent Daily Dose; MEDD) were conducted with time (enrollment, before class, 3-month, 6-month) as a predictor. We used lmer in R (Bates, Mächler, Bolker, & Walker, 2015) to run the multilevel linear models and glmer in R to run the negative binomial model. For each model we included an optimizer (“bobyqa”) and a random intercept. Below are the model formulas:

*Predicting average pain intensity across four timepoints*

**1) AvgPain_ij_  = (**g**_00_ +** g**_10_Time_j_ + u_j)_**

*Predicting pain catastrophizing across four timepoints*

**2) PainCatastrophizing_ij_  = (**g**_00_ +** g**_10_Time_j_ + u_j)_**

*Predicting pain interference across four timepoints*

**3) PainInterference_ij_  = (**g**_00_ +** g**_10_Time_j_ + u_j)_**

*Predicting opioid dose (MEDD) across four timepoints*

**4) MEDD_ij_  = 1 = (**g**_00_ +**  g**_10_Time_j_ + u_j_)**

**1 –MEDD_ij_ = 1**

Across the daily surveys a total of four linear multi-level models were conducted to examine two outcomes, change in average pain intensity and percent change in opioid dose. Change in average pain intensity was baseline calculated (i.e., each daily value subtracted pain intensity values at enrollment), such that negative scores indicated reductions in pain and positive scores indicated an increase in pain relative to enrollment. Percent change in opioid dose was calculated as: $\frac{Daily MEDD-Baseline MEDD}{Baseline MEDD}*100$, such that negative scores indicated a percentage reduction in MEDD and positive scores indicated a percentage increase in MEDD relative to enrollment. Due to outliers at the upper 95% confidence interval bound (80-607% change in MEDD), outlier cases were winsorized to the 95^th^ percentile (77% change in MEDD) to limit distribution skew. A total of 77 out of 1,136 cases (7%) were winsorized. The first two linear multi-level models used the baseline and follow-up daily assessments (before and after class; n = 1,136) to examine predictors of change in average pain intensity and percent change in opioid dose. Below are the model formulas we specified using lmer in R (Bates, Mächler, Bolker, & Walker, 2015) to run the multilevel linear models:

*Predicting change in average pain intensity from enrollment to each daily assessment*

**5) AvgPain_ij_  = (**g**_00_ +** g**_10_PainCatastrophizing_ij_ +**

g**_10_Pre-Post_ij_ +** g**_10_PRN_Cohort_ij +_** g**_10_LA_Cohort_ij +_** g**_10_Bup_Cohort_ij +_** g**_10_ITP_Cohort_ij_** g**_01_PainCatastrophizing*Pre-Post +** g_0_**_1_PRN_Cohort*Pre-Post _+_** g**_01_LA_Cohort*Pre-Post _+_** g**_01_Bup_Cohort*Pre-Post _+_** g**_01_ITP_Cohort*Pre-Post + u_j_)**

*Predicting percent change in opioid dose (Morphine Equivalent Daily Dose; MEDD) from enrollment to each daily assessment*

**6) MEDD_ij_ = (**g**_00_ +** g**_10_PainCatastrophizing_ij_ +** g**_10_AvgPain_ij +_**

g**_10_Pre-Post_ij_ +** g**_10_PRN_Cohort_ij +_** g**_10_LA_Cohort_ij +_** g**_10_Bup_Cohort_ij +_** g**_10_ITP_Cohort_ij_** g**_01_PainCatastrophizing*Pre-Post +** g**_01_AvgPain*Pre-Post +** g_0_**_1_PRN_Cohort*Pre-Post _+_** g**_01_LA_Cohort*Pre-Post _+_** g**_01_Bup_Cohort*Pre-Post _+_** g**_01_ITP_Cohort*Pre-Post + u_j_)**

The final two linear multi-level models used only the follow-up daily assessments (after class; n = 519 surveys; restricted to 41 individuals with at least one data point in follow-up daily assessments). We examined the same factors in the prior models, in addition to use of behavioral skills, to predict change in average pain intensity and percent change in opioid dose. Below are the formulas for the models we ran using the follow-up daily surveys:

*Predicting change in average pain from enrollment to each follow up (after class) daily assessment*

**7) AvgPain_ij_  = (**g**_00_ +** g**_10_PainCatastrophizing_ij_ +**

g**_10_Time_ij_ +** g**_10_Relax_ij_ +** g**_10_Reframe_ij_ +** g**_10_Activities_ij_** + g**_10_PRN_Cohort_ij +_** g**_10_LA_Cohort_ij_** g**_01_PainCatastrophizing*Time +** g_0_**_1_Relax*Time +** g_0_**_1_Reframe*Time _+_** g**_01_Activities *Time _+_** g_0_**_1_Relax*PRN +** g_0_**_1_Reframe*PRN _+_** g**_01_Activities *PRN _+_** g_0_**_1_Relax*LA +** g_0_**_1_Reframe*LA _+_** g**_01_Activities *LA + u_j_)**

*Predicting percent change in opioid dose (MEDD) from enrollment to each follow up (after class) daily assessment*

**8) MEDD_ij_  = (**g**_00_ +** g**_10_PainCatastrophizing_ij_ +** g**_10_AvgPain_ij_ +**

g**_10_Time_ij_ +** g**_10_Relax_ij_ +** g**_10_Reframe_ij_ +** g**_10_Activities_ij_** + g**_10_PRN_Cohort_ij +_** g**_10_LA_Cohort_ij_** g**_01_PainCatastrophizing*Time +** g**_01_AvgPain*Time +** g_0_**_1_Relax*Time +** g_0_**_1_Reframe*Time _+_** g**_01_Activities *Time _+_** g_0_**_1_Relax*PRN +** g_0_**_1_Reframe*PRN _+_** g**_01_Activities *PRN _+_** g_0_**_1_Relax*LA +** g_0_**_1_Reframe*LA _+_** g**_01_Activities *LA + u_j_)**
