# Supplementary material for: Zoom-Delivered Empowered Relief for Chronic Pain: Observational Longitudinal Pilot Study Exploring Feasibility and Pain-Related Outcomes in Patients on Long-Term Opioids
Source: JMIR Form Res. 2025 Mar 11;9:e68292. doi: 10.2196/68292 (PMC11937707; doi:10.2196/68292)
Supplement: Multimedia Appendix 3 [file formative_v9i1e68292_app3.docx]

**Multimedia Appendix 3**

Supplemental Table 1. Type of prescribed opioid used at enrollment.

| Opioid Type^*^ | N | % |
| --- | --- | --- |
| Oxycodone | 15 | 24% |
| Norco | 11 | 18% |
| Tramadol | 9 | 15% |
| Suboxone/buprenorphine | 8 | 13% |
| Hydrocodone | 6 | 10% |
| Other^a^ | 6 | 10% |
| Dilaudid/Hydromorphone | 4 | 6% |
| MS Contin | 4 | 6% |
| Morphine | 4 | 6% |
| Percocet | 3 | 5% |
| Oxycontin | 2 | 3% |
| Methadone | 2 | 3% |
| Vicodin | 1 | 2% |
| Fentanyl | 1 | 1% |

^*^Could select multiple categories. ^a^ = Gralise, Belbucca, Xtampza, Nucenta.
