# Supplementary material for: Zoom-Delivered Empowered Relief for Chronic Pain: Observational Longitudinal Pilot Study Exploring Feasibility and Pain-Related Outcomes in Patients on Long-Term Opioids
Source: JMIR Form Res. 2025 Mar 11;9:e68292. doi: 10.2196/68292 (PMC11937707; doi:10.2196/68292)
Supplement: Multimedia Appendix 4 [file formative_v9i1e68292_app4.docx]

**Multimedia Appendix 4**

Supplemental Table 2. A sensitivity analysis of multi-level regressions examining changes in pain and opioid outcomes across monthly assessments without Buprenorphine, Intrathecal Pump, Methadone, and Tramadol users included.

|  | **Outcome** | **Variables** | **Coefficient^a^** | **SE** | **Test Statistic^a^** | ***p*** |
| --- | --- | --- | --- | --- | --- | --- |
| Linear | Pain Intensity | *Intercept* | 5.87 | .24 | 24.54 | < .001^*^ |
|  |  | *Pre-class*^b^ | -.26 | .21 | -.84 | .22 |
|  |  | *3-months*^b^ | -.59 | .24 | -2.67 | .01 |
|  |  | *6-months*^b^ | -.33 | .25 | -1.37 | .19 |
|  | Pain Interference | *Intercept* | 62.75 | 1.24 | 50.44 | < .001^*^ |
|  |  | *Pre-class*^b^ | -0.96 | .88 | -1.10 | .27 |
|  |  | *3-months*^b^ | -2.00 | 1.00 | -1.99 | .05 |
|  |  | *6-months*^b^ | -1.86 | 1.04 | -1.78 | .08 |
|  | Pain  Catastrophizing | *Intercept* | 16.54 | 1.60 | 10.33 | < .001^*^ |
|  |  | *Pre-class*^b^ | -2.59 | 1.31 | -1.97 | .05 |
|  |  | *3-months*^b^ | -3.84 | 1.51 | -2.56 | .01^*^ |
|  |  | *6-months*^b^ | -3.28 | 1.56 | -2.09 | .04 |
| Negative Binomial | Opioid Dose (MEDD) | *Intercept* | 39.2 | 6.04 | 23.8 | < .001^*^ |
|  |  | *Pre-class*^b^ | 0.99 | .08 | -.03 | .85 |
|  |  | *3-months*^b^ | 0.86 | .08 | -1.61 | .10 |
|  |  | *6-months*^b^ | .91 | .09 | -.90 | .37 |

^a^ The linear multilevel model reports a beta coefficient, and t-value statistics. The negative binomial multilevel model, via glmer, reports incidence rate ratios (IRRs) and z-value statistics.

^b^ Reference group is baseline. ^c^ Effect size *d* calculated as baseline minus follow-up divided by pooled SD. Positive *d* values indicate improvements. ^*^Significant based on corrected p-value.
