# Supplementary material for: Zoom-Delivered Empowered Relief for Chronic Pain: Observational Longitudinal Pilot Study Exploring Feasibility and Pain-Related Outcomes in Patients on Long-Term Opioids
Source: JMIR Form Res. 2025 Mar 11;9:e68292. doi: 10.2196/68292 (PMC11937707; doi:10.2196/68292)
Supplement: Multimedia Appendix 5 [file formative_v9i1e68292_app5.docx]

**Multimedia Appendix 5**

Supplemental Table 3. A sensitivity analysis of multi-level linear regressions predicting change in pain intensity and opioid dose from baseline across the baseline (before class) and follow-up (after class) daily dairies without Buprenorphine, Intrathecal Pump, Methadone, and Tramadol users included.

| **Outcome** | **Variables** | **Coefficient^a^** | **SE** | **T** | ***p*** |
| --- | --- | --- | --- | --- | --- |
| Change in  Pain Intensity^b^ | *Intercept* | -.65 | .54 | -1.22 | .23 |
|  | *PCS^d^* | .41 | .03 | 16.10 | < .001^*^ |
|  | *Pre-Post^e^* | -.41 | .13 | -3.16 | .002 |
|  | *PRN Cohort* | -.22 | .64 | -.35 | .73 |
|  | *LA Cohort* | -.02 | .71 | -.03 | .98 |
|  | *Pre-Post x PCS* | .04 | .04 | .86 | .39 |
|  | *Pre-Post x PRN* | .13 | .17 | .78 | .43 |
|  | *Pre-Post x LA* | -.77 | .20 | -3.91 | < .001^*^ |
| Percent change in  Opioid Dose^c^ | *Intercept* | 3.21 | 11.63 | .27 | .78 |
|  | *PCS^d^* | 2.64 | .90 | 2.93 | .003^*^ |
|  | *Pre-Post^e^* | 1.57 | 3.61 | .43 | .66 |
|  | *Avg Pain* | 3.58 | 1.40 | 2.56 | .01 |
|  | *PRN Cohort* | -31.2 | 13.83 | -2.26 | .03 |
|  | *LA Cohort* | -13.92 | 15.39 | -0.90 | .37 |
|  | *Pre-Post x PCS* | -1.61 | 1.49 | -1.08 | .28 |
|  | *Pre-Post x Pain* | -1.59 | 2.34 | -.68 | .50 |
|  | *Pre-Post x PRN* | -10.69 | 4.68 | -2.29 | .02 |
|  | *Pre-Post x LA* | 4.19 | 5.60 | .75 | .45 |

^a^Unstandardized beta coefficient. ^b^Outcome is change in average pain intensity in the past 24 hours and depicts change at each daily assessment compared to enrollment. ^c^Percent change in opioid dose is measured using morphine equivalent daily dosage (MEDD) and depicts change at each daily assessment compared to enrollment. ^d^Daily pain catastrophizing scale. ^e^ 0 = pre-class daily assessments, 1 = post-class daily assessments. ^*^Significant based on corrected *p*-value. PRN = short acting opioids as needed; LA = long acting opioids.
