# Supplementary material for: Zoom-Delivered Empowered Relief for Chronic Pain: Observational Longitudinal Pilot Study Exploring Feasibility and Pain-Related Outcomes in Patients on Long-Term Opioids
Source: JMIR Form Res. 2025 Mar 11;9:e68292. doi: 10.2196/68292 (PMC11937707; doi:10.2196/68292)
Supplement: Multimedia Appendix 6 [file formative_v9i1e68292_app6.docx]

**Multimedia Appendix 6**

Supplemental Table 4. Multi-level linear regressions predicting change in pain intensity and opioid dose from enrollment across the follow-up (after class) daily dairies.

| **Outcome** | **Variables** | **Coefficient^a^** | **SE** | **T** | ***p*** |
| --- | --- | --- | --- | --- | --- |
| Change in  Pain Intensity^b^ | *Intercept* | -2.27 | .20 | -1.40 | .17 |
|  | *PCS^d^* | .48 | .04 | 12.24 | < .001^*^ |
|  | *Time^e^* | -.01 | .01 | -1.22 | .22 |
|  | *Relax* | .67 | .31 | 2.17 | .03 |
|  | *Reframe* | -.27 | .33 | .83 | .41 |
|  | *Actions* | -.42 | .36 | -1.19 | .24 |
|  | *PRN Cohort* | .13 | .21 | .64 | .53 |
|  | *LA Cohort* | -.04 | .25 | -.17 | .87 |
|  | *Time x PCS* | -.01 | .01 | -1.84 | .07 |
|  | *Time x Relax* | -.08 | .03 | -2.74 | .006 |
|  | *Time x Reframe* | .02 | .03 | .73 | .47 |
|  | *Time x Actions* | -.01 | .03 | -.06 | .95 |
|  | *Relax x PRN* | .07 | .26 | .26 | .79 |
|  | *Relax x LA* | -.16 | .36 | -.48 | .62 |
|  | *Reframe x PRN* | -.27 | .28 | -.97 | .33 |
|  | *Reframe x LA* | .60 | .38 | 1.58 | .12 |
|  | *Actions x PRN* | .46 | .30 | 1.53 | .13 |
|  | *Actions x LA* | .52 | .39 | 1.32 | .19 |
| Percent change in opioid dose^c^ | *Intercept* | -8.17 | 12.83 | -.64 | .53 |
|  | *PCS^d^* | .70 | 1.45 | .59 | .63 |
|  | *Avg Pain* | .97 | 2.15 | .45 | .65 |
|  | *Time^e^* | .28 | .26 | 1.06 | .29 |
|  | *Relax* | 14.78 | 8.50 | 1.74 | .09 |
|  | *Reframe* | -8.25 | 8.84 | -.93 | .35 |
|  | *Actions* | 7.63 | 9.69 | .79 | .43 |
|  | *PRN Cohort* | -8.37 | 14.65 | -.57 | .57 |
|  | *LA Cohort* | -.26 | 17.43 | -.02 | .99 |
|  | *Time x PCS* | .05 | .17 | .26 | .79 |
|  | *Time x Avg Pain* | .26 | .27 | .97 | .34 |
|  | *Time x Relax* | -1.15 | .83 | -1.38 | .17 |
|  | *Time x Reframe* | .91 | .88 | 1.03 | .30 |
|  | *Time x Actions* | -.63 | .92 | -.69 | .49 |
|  | *Relax x PRN* | 7.10 | 6.88 | 1.03 | .30 |
|  | *Relax x LA* | -10.37 | 9.82 | -1.06 | .29 |

(continued on next page)

Supplemental Table 4. (continued)

| **Outcome** | **Variables** | **Coefficient^a^** | **SE** | **T** | ***p*** |
| --- | --- | --- | --- | --- | --- |
| Percent change in opioid dose^c^ | *Reframe x PRN* | .85 | 7.44 | .12 | .91 |
|  | *Reframe x LA* | 8.98 | 10.27 | .88 | .38 |
|  | *Actions x PRN* | -4.82 | 8.13 | -.59 | .55 |
|  | *Actions x LA* | -15.04 | 10.71 | -1.40 | .16 |

^a^Unstandardized beta coefficient. ^b^Change in average pain intensity in the past 24 hours and depicts change at each daily assessment compared to enrollment (e.g., negative value means pain decreased compared to enrollment). ^c^Percent change in opioid dose is measured using morphine equivalent daily dosage (MEDD) and depicts change at each daily assessment compared to enrollment (e.g., negative value means opioid dose decreased compared to enrollment). ^d^Daily pain catastrophizing scale. ^e^Daily assessment. ^*^Significant based on corrected *p*-value. PRN = short acting opioids as needed; LA = long acting opioids.
